# Does Endoscopic Transnasal Optic Nerve Decompression Followed by Radiosurgery Improve Outcomes in the Treatment of Parasellar Meningiomas?

**DOI:** 10.3390/medicina58081137

**Published:** 2022-08-22

**Authors:** Petr Matoušek, Jakub Cvek, Lenka Čábalová, Eva Misiorzová, Ondřej Krejčí, Radim Lipina, Tomáš Krejčí

**Affiliations:** 1 Department of Otorhinolaryngology, Head and Neck Surgery, University Hospital Ostrava, 70852 Ostrava, Czech Republic; 2 Faculty of Medicine, University of Ostrava, 70300 Ostrava, Czech Republic; 3 Department of Oncology, University Hospital Ostrava, 70852 Ostrava, Czech Republic; 4 Department of Neurosurgery, University Hospital Ostrava, 70852 Ostrava, Czech Republic

**Keywords:** skull base surgery, endoscopic endonasal surgery, optic nerve decompression, parasellar meningioma, stereotactic radiosurgery

## Abstract

Introduction: The clinical management of parasellar meningiomas (PM) is challenging due to their intimate association with critical neurovascular structures. Consensus regarding the recommended treatment protocol is lacking. This study will evaluate patients’ visual outcomes following endoscopic transnasal optic nerve decompression (ETOND) and will investigate the possibility of reducing the rate of complications associated with stereotactic radiosurgery (SRS). Methods: Retrospective analysis was conducted on all patients who underwent ETOND for PM between 2013 and 2020. The study comprised 12 patients (7 women and 5 men aged 36–75 years; mean, 55.2 years; median, 57.6 years) in which 14 optic nerve decompression procedures were carried out. Patients were followed up for 6 to 86 months (mean, 29.3 months; median, 25 months). There were five cases of spheno-orbital meningioma, four cases of cavernous sinus meningioma, and one case each of petro-clival meningioma, optic nerve sheath meningioma, and planum sphenoidale/tuberculum sellae meningioma. Visual outcome was evaluated and any postoperative complications noted. Results: Improvements in visual acuity were noted in 10 of 14 eyes (71.4%) 3 to 6 months postoperation. Visual acuity remained stable in the remaining four eyes. No deterioration of visual acuity was noted during the follow-up period. In total, 9 of the 12 patients underwent SRS. No tumor growth was determined, while reduction in tumor volume was noted in five patients following SRS. No complications associated with SRS or the surgical procedure were noted. Conclusions: ETOND appears to be a promising technique for increasing rates of improved visual function, while reducing the risk of post SRS-related complications. In combination with subsequent SRS, it is an ideal treatment modality in the management of parasellar meningiomas. Confirmation of our findings would require a larger, prospective multicenter study.

## 1. Introduction

As a junction of important neurovascular structures, the parasellar region is anatomically complex. Meningiomas are one of the most frequent tumors arising in this area [1]. Although these are mostly WHO Grade I, their location often makes them hard to reach, hindering their safe and complete resection by microsurgical or endoscopic techniques [1,2,3,4]. The management of PM thus remains controversial. The most frequent symptom of such tumors is visual impairment caused by compression of the optic nerve (ON) or chiasma. [1]. Optic nerve compression is typically caused by compression in the optic canal directly at the site of tumor growth or, less commonly, by excrescence from the optic canal. Surgical decompression of the ON followed by stereotactic radiosurgery (SRS) is a treatment option in these cases. SRS is increasingly employed as the first-line treatment [2,5,6,7], characterized by good long-term lesion control, although with relatively low rates of improvement of neurological symptoms and the risk of post SRS-related complications [2,6,8]. Endoscopic transnasal optic nerve decompression (ETOND) followed by SRS appears to be an alternative to transcranial surgical approaches. Although only sporadically published, studies of ETOND for slow-growing lesions and meningiomas describe favorable outcomes in terms of postoperative visual improvement [9,10,11,12,13]. This minimally invasive technique allows excellent visualization of the course of the optic nerve and orbital apex. In this study, we retrospectively evaluate our patients’ visual outcomes following ETOND and SRS.

## 2. Patients and Methods

### 2.1. Methods

Retrospective analysis was conducted on all patients who underwent ETOND for a PM invading the optic canal between 2013 and 2020. Data were gathered from clinical documentation, including the results of pre- and postoperative ophthalmological examination, surgical protocols, and imaging studies. The visual outcome was evaluated at 3 to 6 months after ETOND and over the remainder of the follow-up period. SRS was performed with the CyberKnife system. The effect of SRS on visual outcome was also evaluated. All patients underwent ophthalmological investigation pre-operatively at 3 to 6 months postoperation and subsequently once every 6 to 12 months. Visual acuity was assessed with the Snellen visual acuity test and visual field testing. Improved visual acuity was defined as at least a 2-line improvement in reading ability on visual acuity testing. Included in the analysis were any surgical complications, along with radiological findings of the monitored meningiomas. Post SRS, reductions in tumor volume of at least 10% were attributed to the procedure. Reductions in tumor volume were measured using an open-source DICOM viewer (Medixant, Poznan, Poland).

### 2.2. Patient Characteristics

A total of 12 patients (7 women and 5 men aged 36–75 years; mean, 55.2 years; median, 57.6 years) underwent a total of 14 optic nerve decompression procedures (9 right- and 5 left-sided). Patients were followed up for 6 to 86 months (mean, 29.3 months; median, 25 months).

Optic canal invasion by meningioma—the indication for ETOND—was present in all patients. ETOND was carried out in combination with the endoscopic extended transnasal approach (EETA) in two cases, transnasal resection of the extradural portion in two cases, and endoscopic transorbital resection in one case. In the case of follow-up EETA, early ETOND was performed as the primary operation. Biopsies were performed during another four ETONDs and submitted for histology. There were five cases of spheno-orbital meningioma, four cases of cavernous sinus meningioma, and one case each of petro-clival meningioma, optic nerve sheath meningioma, and planum sphenoidale/tuberculum sellae meningioma. Meningiomas were histopathologically confirmed as WHO Grade I in nine cases and Grade III in one case. Tissue samples were not obtained for histopathology in two cases.

A total of 7 patients in the study group had undergone at least 1 previous transcranial microsurgical procedure. In total, 9 out of 12 patients underwent follow-up SRS, in all cases within 3 months of ETOND.

Progressive visual impairment ipsilateral to ON compression was observed in 10 of 12 patients (83%). Two patients were indicated for ETOND despite exhibiting no signs of visual impairment because of optic canal invasion and with regard to the planned SRS. Blindness in the other, unoperated eye was determined in two patients. Cranial nerve palsy was observed in 4 of 12 patients (33.3%), most frequently in CN III and V.

### 2.3. Surgical Technique

**Figure 1:**
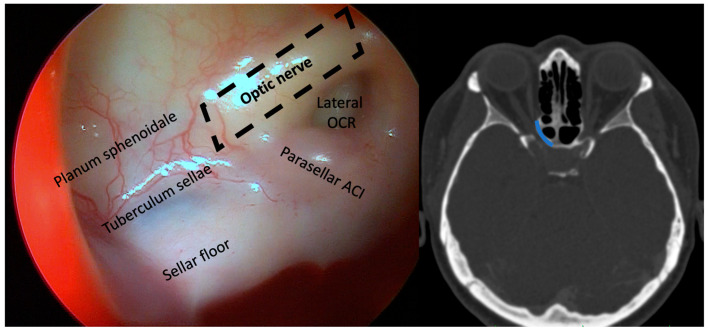
**Left**: sphenoid sinus view of the left ON and parasellar internal carotid artery. The dotted line outlines the optic canal. **Right**: axial CT imaging in bone window. The line highlights the extent of optic canal decompression.

**Figure 2:**
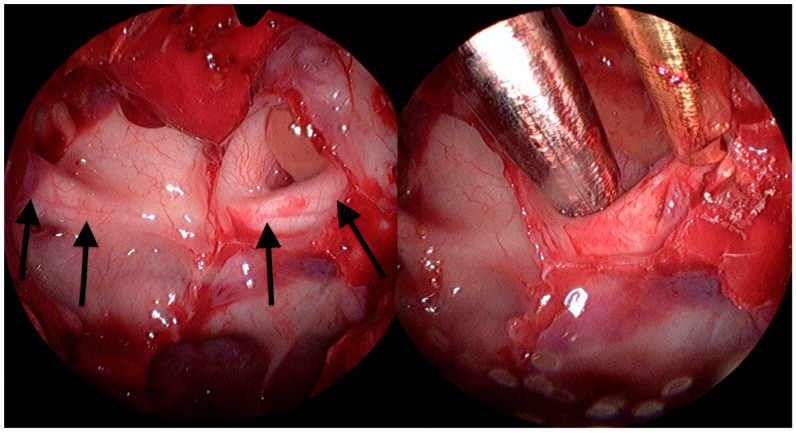
Sphenoidal sinus view. The image **left** shows prominence of both optic nerves (arrows). **Right**: decompression of the left ON is shown.

By a transnasal endoscopic approach (MINOP TREND, Aesculap, Center Valley, PA, USA), the sphenoid sinus was opened widely, and a posterior septectomy performed to visualize the lateral course of the optic nerve (Figure 1). Following ethmoidectomy, the lamina papyracea was exposed in the posterior ethmoid sinus and resected with elevators in the orbital apex at the level of the annulus of Zinn. The bone dorsal to the optic nerve protuberance was first thinned with a diamond burr and then removed with Kerrison rongeurs and elevators from the dorsal sphenoid sinus wall up to the intracranial ON segment (Figure 2 and Video S1). A binostril approach with both sphenoid sinuses opened was always employed, thus enabling multihand microsurgical techniques and irrigation, while drilling near the ON. Nerve sheath incision was not performed.

## 3. Results

Patient outcome and characteristics are summarized in Table 1. Improved visual function was defined as at least a 2-line improvement in reading ability. Improved visual acuity was noted in 10 of 14 eyes (71.4%) in 8 of 12 patients (66.7%) at the initial postoperative follow-up at 3 months postoperation. In two patients (2 eyes) with pre-operative visual impairment, postoperative stabilization of visual acuity was noted, remaining so over the monitoring period. Visual acuity remained stable in two patients (2 eyes) presenting with no signs of visual impairment pre-operatively. There were no cases of ETOND-related visual impairment postoperatively, nor were any such cases noted during the follow-up period (mean, 29.3 months; median, 25 months). No surgery-related complications were noted.

A total of 9 out of 12 patients underwent SRS (see Table 2). In all cases this was performed within 3 months of ETOND by a robotic radiotherapy device (CyberKnife, Sunnyvale, CA, USA). Repeat SRS was performed in one patient (Patient 11), who had initially undergone transcranial surgery. Transcranial surgery and subsequent SRS were planned for Patient 3, who refused further treatment; her clinical and graphical state remains stable and without progression. Patient 4 was indicated for SRS, which was postponed due to ongoing treatment for breast carcinoma. The patient was lost to monitoring after 6 months. Patient 6 was postoperatively diagnosed with progressive bulbar palsy and was not inclined to undergo radiosurgery because of the risk of exacerbating cranial nerve paresis. The patient was lost to monitoring after 6 months.

Following SRS, tumor volume was reduced in five patients and stable in four patients at 6 months postoperation, with no progression observed over the follow-up period. No complications in terms of new cranial nerve palsy or visual impairment were reported following the procedure. Tumor volume before and after surgery is summarized in Table 3.

### Illustrative Case Report

**Figure 3:**
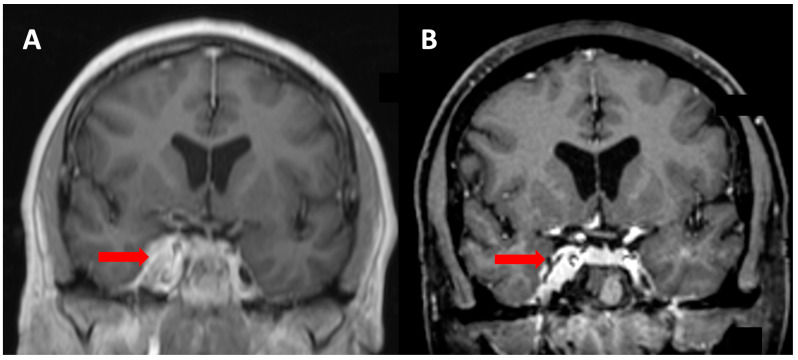
Meningeoma (red arrow) of Patient 1 infiltrating the cavernous sinus and optic canal with encasement of the right internal carotid artery (**A**). Tumor volume regression of 28% noted at final MRI follow-up (**B**).

**Figure 4:**
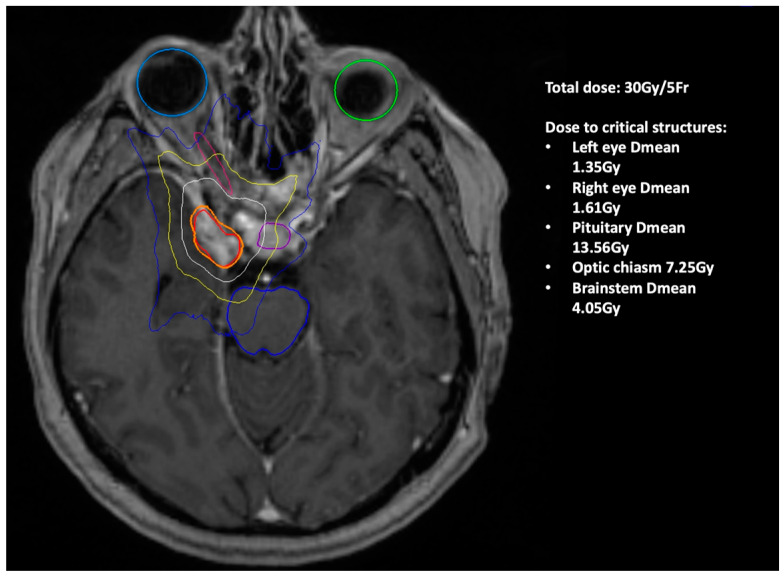
Illustration of the irradiated SRS field (Patient 1). The colored circles show isodose—areas with the same dose of radiation (orange is the planned dose of 30 Gy, red is the maximum dose, white is 20 Gy dose area, yellow 10 Gy, blue 5 Gy). Light blue circle shows the right eye, green the left. The pituitary gland is shown in purple and the brain stem in blue.

A 60-year-old female was referred from ophthalmology with a half-year history of deteriorating vision and abnormal visually evoked potentials and perimetry in the right eye. Best-corrected visual acuity was 20/400. Other cranial nerves were free of clinical symptoms. Brain MRI revealed a meningioma of the right cavernous sinus with infiltration of the optic canal and right carotid artery encasement (Figure 3). The tumor volume was 5.13 cm^3^. Radical extirpation was not possible. Given the findings and symptoms, the patient was indicated for transnasal endoscopic decompression of the optic canal followed by radiosurgery. During the surgical procedure the ethmoid and sphenoid sinuses were opened. Decompression of the orbital apex and optic canal up to the planum sphenoidale was performed using microdissection and Kerrisons. Bone from the medial orbital wall was sent for histology, which confirmed a WHO Grade I meningioma. The procedure took 92 min and no complications were noted(see Video S1 in Supplementary Materials Section). The patient underwent radiosurgery 9 weeks postoperation (Figure 4), with significant improvement in visual acuity confirmed at ophthalmological follow-up 3 months postoperation. Vision remained stable during the follow-up period (54 months), during which time the tumor gradually regressed by 28%.

## 4. Discussion

Treatment of a PM is challenging and requires cross-disciplinary cooperation for an optimal outcome. Only some centers can guarantee radical and safe resection of complex midline skull base meningiomas [4,14]. Appropriate surgical techniques are repeatedly discussed and are precisely summarized in Mastantuoni et al. [3]. Unlike other meningiomas, complete resection, although the ideal, is not usually possible in these cases; rather, the aim of treatment, in addition to long-term growth control, is improvement or preservation of neurological function. Aggressive resection, which involves a relatively high risk of permanent neurological deficit or death in such cases [15], has been abandoned in favor of a more circumspect approach aiming to safely reduce tumor volume and decompress neurological structures with follow-up SRS [16,17,18]. This is the strategy we subscribe to. Given that visual impairment is one of the most common symptoms [1] and that optic canal invasion is determined in the majority of these cases [9], it is logical to attempt to preserve visual function with optic canal decompression and, where indicated, safely reduce tumor volume. A range of ON decompression modalities have been discussed ever since the possibility of such a procedure was first published in the 1960s [19,20]. Nowadays, endoscopic transnasal techniques are becoming the preferred modality for reaching skull base lesions [17,21]. Such techniques enable excellent visualization of the optic nerve and orbital apex with minimal invasivity. According to an anatomical study involving 10 ON decompressions, ETOND enables sufficient optic canal decompression (an average of 168°) [7]. The advantage of this technique is that any experienced skull base surgeon will be well acquainted with it. On the other hand, the associated learning curve presents a certain disadvantage at an inexperienced center, material requirements notwithstanding. Otherwise, the technique is easily mastered and does not differ from conventional transnasal endoscopic surgery. We also consider it an advantage that optic canal decompression can be supplemented by guided EETA for reducing the intradural portion of the meningioma and allowing further decompression of neural structures. In this study, tumor reduction was performed in 5 of 12 patients; EETA was carried out in 2 of these.

Lund and Rose were the first, in 2006, to describe ETOND for nontraumatic optic neuropathy due to meningioma invading the optic canal [11]. Since then, several other papers have described good visual outcome with ETOND in patients with nontraumatic optic neuropathy caused by compression from variously PM, fibro-osseous tumors, inflammatory pseudotumors, and even in cases of idiopathic intracranial hypertension [9,10,11,12,13,19,22,23]. These studies confirm ETOND as a safe and effective modality for the restoration of visual function, with good visual outcomes determined in 55.4% to 100% of cases. We observed improved visual acuity in 71.4% of eyes. No surgery-related complications were noted in our study of 12 patients, corresponding to the experience in the wider literature [1,14,15,16,17,18,19]. Only Berhouma et al. have encountered ETOND-related complications, with 1 case of epistaxis and 1 case of orbital emphysema in a study of 11 patients [19]. The same author also describes worsening visual acuity in 1 patient despite undergoing ETOND—a complication that has not been described by other authors. A higher risk of postoperative complications, most frequently CSF leakage, is associated with patients undergoing ETOND supplemented by EETA. However, the incidence of this complication is falling as the technique is perfected: according to the largest studies, the rate of CSF leak is approximately 16% [21].

For meningiomas in which radical resection is complicated, SRS is increasingly used, either alone or in combination with surgery [2,5,6,8,16]. Most studies report overall tumor control rates of over 90%, although with variable neurological improvement rates of between 8% and 66%, and post SRS complication rates between 3% and 40% [2,6,7,8,24]. In studies of more than 100 patients receiving tumor margin doses of 12 to 15 Gy, rates of complications ranged from 0% to 16% [2]. SRS-induced cranial nerve deficit is most likely in CN II and V. The maximum safe dose to the optic apparatus remains controversial because visual decline has been noted even with low doses (under 8 Gy) [2,6]. In addition to the overall dose, the length of irradiated CN II plays a role [2]. In a series of 189 patients, Cohen-Inbar noted 52 with visual impairment, which worsened in 18 cases (34.6%) post SRS. Improved or stable outcomes are not mentioned [2]. A multicenter study by Sheehan et al. evaluated post SRS outcomes in a representative sample of 763 patients (50.7% of which had undergone at least one resection before SRS) and determined an overall favorable outcome in 79.6% [6]. This conclusion was reached in all patients with no tumor progression, no worsening of a pre-existing CN deficit, and no new neurological deficit. No difference in overall outcome was observed between patients who underwent resection prior to SRS (the type of operation was not specified) and those who underwent primary SRS [6]. Prior CN deficit was observed in 529 patients of which 99 had visual impairment. Post SRS, just 179 of these patients (34%) demonstrated improvement, while visual acuity improved in only 22 of the 99 with pre-operative visual impairment [6]. New or worsening CN deficits were observed in 9.6% of patients [6]. Although the overall tumor control rate was 90.2% over a median follow-up period of 66.7 months, the question is whether this can be considered successful given that more significant improvements in neurological function could be achieved using a strategy of safe tumor reduction and decompression of neural structures. Lee et al. have discussed ‘radiosurgical decompression’ for compressive cranial neuropathies [5]. After hypofractionated SRS, vision improved in 21 of 38 eyes (55.3%), was unchanged in 14 (36.8%), and worsened in 3 (7.9%) [5]. Two of these three eyes deteriorated because of transient tumor swelling after SRS [5]. In these cases, widening the optic canal by ETOND would seem to be indicated, enabling safe tumor reduction and decompression of optical apparatus, as recommended elsewhere [12,16,17,19], which should prevent post-SRS complications and lead to a greater chance of restoring neurological function. We encountered no post SRS complications in terms of worsening CN function during the monitoring period.

An alternative to prevent postradiation damage to the optic nerve can be the insertion of a fat graft between the nerve and the rest of the tumor, as reported by Starnoni et al. [18]. We consider the risks associated with open surgery and the resorption of the fat graft over time to be the disadvantages of this technique.

SRS provides excellent long-term management of PMs, although surgery still has its place in their treatment. It is necessary to consider the benefits of both modalities for optimal treatment outcomes. Given the high rate of visual impairment in such cases, we believe ETOND (with tumor reduction where indicated) in combination with subsequent SRS to be the treatment of choice. In our experience this approach offers a greater likelihood of improved visual function, while reducing post SRS complications. A more extensive, prospective study with long-term patient monitoring is required to confirm our findings.

## 5. Conclusions

As a treatment modality for enhancing rates of improved visual function while also reducing the risk of postoperative SRS-related complications, ETOND appears promising. In combination with subsequent SRS it is an ideal treatment modality in the management of parasellar meningiomas. We recommend this strategy in cases where there are doubts regarding the safety or feasibility of radical resection. Larger prospective and multicenter study is necessary to confirm our findings.

## Figures and Tables

**Table 1:** Patient characteristics and visual outcome.

							Best-Corrected Visual Acuity						
Case Number	Sex/Age	Meningioma Type	Who Grade	Eye	Associated Surgery Type	Previous Transcran. Surgery	Preoperative	Postoperative	Postop. Visual Field Test	Other Cranial Nerves Lesion	Srs *	Surgical Complications	Follow-Up (Months)
1.	F/60	Cavernous sinus	I	right	biopsy	No	20/400	20/50	improved	None	Yes	None	54
2.	M/66	Petroclival	I	right	extradural portion resection	Yes	20/200	20/200	stable	None	Yes	None	22
3.	F/57	Spheno-orbital	I	left	transorbital, endoscopic resection	No	20/30	20/30	normal	None	No	None	31
4.	F/66.5	Spheno-orbital	I	right	biopsy	Yes	20/20	20/20	normal	Blindness, CN III, VI left	No	None	6
5.	M/46.5	Tuberculum sellae and planum sphenoidale	I	bilaterally	extended approach	Yes	Right: 20/100 Left: 20/200	Right: 20/20 Left: 20/50	improved	None	Yes	None	14
6.	F/75	Spheno-orbital	I	left	extradural portion resection	Yes	Hand motion	20/100	improved	CN V left	No	None	6
7.	M/58	Cavernous sinus	I	left	none	Yes	Finger count/20 cm	20/70	improved	None	Yes	None	23
8.	M/59	Optic nerve sheat	N/A	right	none	No	20/200	20/70	improved	None	Yes	None	27
9.	F/44	Spheno-orbital	I	bilaterally	none	Yes	Right: 20/200 Left: finger count/20 cm	Right: 20/70 Left: 20/100	improved	None	Yes	None	34
10.	F/39	Spheno-orbital	N/A	right	biopsy	No	20/100	20/40	improved	CN III, VI right	Yes	None	28
11.	F/36	Cavernous sinus	III	right	extended approach	Yes	20/70	20/30	improved	Blindness, V left	Yes	None	86
12.	M/55	Cavernous sinus	I	right	biopsy	No	20/50	20/50	stable	CN III, V right	Yes	None	21

* SRS, Stereotactic Radiosurgery.

**Table 2:** CyberKnife radiosurgery—treatment parameters and patient outcome.

Case Number	Sex/Age	Dosage (gy)	Fractions	PTV (cm^3^)	Isodose	Associated Complications (CN Deficit)	Tumor Response
1.	F/60	30	5	5.13	66	none	regression
2.	M/66	25	5	33.47	61	none	stable
5.	M/46.5	30	6	9.3	71	none	regression
7.	M/58	30	5	6.41	65	none	stable
8.	M/59	25	5	0.521	74	none	regression
9.	F/44	30	5	5.96	61	none	stable
10.	F/39	30	5	17.01	65	none	stable
11.	F/36	80	5	11.6	82	none	regression (after last SRS)
			3	15.84	62		
12.	M/55	30	5	7.65	73	none	regression

PTV, planning target volume; CN, cranial nerves.

**Table 3:** Tumor volume and development over the monitoring period.

Case Number	Tumor Volume Pre Etond (cm^3^)	Associated Surgery Type	Tumor Volume Pre Srs/Post-Surgery (cm^3^)	End of Follow-up Volume (cm^3^)	Follow-Up (Months)
1.	5.13	biopsy	5.13	3.69	54
2.	37.82	extradural portion resection	33.47	32.9	22
3.	24.8	transorbital, endoscopic resection	17.2	17.1	31
4.	16.56	biopsy	16.56	16.56	6
5.	12.8	extended approach	9.3	5.4	14
6.	18.8	extradural portion resection	15.4	15.4	6
7.	6.41	none	6.41	6.11	23
8.	0.521	none	0.521	0.42	27
9.	5.96	none	5.96	5.9	34
10.	17	biopsy	17.01	17	28
11.	19.31	extended approach	15.84	11.8	86
12.	7.6	biopsy	7.65	6.2	21

## Data Availability

At the request of the corresponding author.

## Supplementary Materials

The following supporting information can be downloaded at: https://www.mdpi.com/article/10.3390/medicina58081137/s1, Video S1: Demonstration of endoscopic transnasal optic nerve decompression.
